# Особенности репликативных и биохимических аспектов старения у женщин при различных формах неятрогенного гипергонадотропного гипогонадизма

**DOI:** 10.14341/probl13295

**Published:** 2023-06-30

**Authors:** Р. К. Михеев, Е. Н. Андреева, О. Р. Григорян, Е. В. Шереметьева, Ю. С. Абсатарова, Н. Н. Волеводз, Е. В. Логинова

**Affiliations:** Национальный медицинский исследовательский центр эндокринологии; Национальный медицинский исследовательский центр эндокринологии; Московский государственный медико-стоматологический университет имени А.И. Евдокимова; Национальный медицинский исследовательский центр эндокринологии; Национальный медицинский исследовательский центр эндокринологии; Национальный медицинский исследовательский центр эндокринологии; Национальный медицинский исследовательский центр эндокринологии; Российский университет дружбы народов

**Keywords:** менопауза, преждевременная недостаточность яичников, теломеры, биохимический анализ, ФСГ

## Abstract

**ОБОСНОВАНИЕ:**

ОБОСНОВАНИЕ. В основе развития возраст-обусловленной патологии у женщин лежит эстрогенный дефицит, приводящий по механизму отрицательной обратной связи к формированию гипергонадотропного гипогонадизма. Существуют небезосновательные предположения о взаимосвязи гипергонадотропного гипогонадизма с репликативными (длина теломер) и биохимическими показателями у женщин с эстрогенным дефицитом как физиологического (менопаузального), так и патологического (преждевременная недостаточность яичников (ПНЯ)) генеза.

**ЦЕЛЬ:**

ЦЕЛЬ. Изучить особенности репликативного клеточного старения (длина теломер) и биохимических показателей у женщин с гипергонадотропным гипогонадизмом физиологического (менопауза) и патологического генеза (ПНЯ).

**МАТЕРИАЛЫ И МЕТОДЫ:**

МАТЕРИАЛЫ И МЕТОДЫ. Исследование проведено на базе ФГБУ «НМИЦ эндокринологии» Минздрава России совместно с МНОЦ «МГУ им. М.В. Ломоносова» в период с 10.01.2021 г. по 01.08.2022 г.

В одномоментном сравнительном исследовании приняли участие 110 женщин (20–75 лет).

**Группа 1:**

Группа 1: 26 женщин, принимавших менопаузальную гормональную терапию (МГТ) ≥5 лет в дозе эстрогенового компонента 1; 2 мг.

**Группа 2:**

Группа 2: 27 участниц с физиологической менопаузой без МГТ.

**Группа 3:**

Группа 3: 33 пациентки с ПНЯ, получавших гормональную заместительную терапию (ГЗТ) половыми стероидами.

**Группа 4:**

Группа 4: 24 здоровых женщины репродуктивного возраста без ГЗТ.

Пациенткам проведены лабораторный генетический (длина теломер лейкоцитов), биохимический анализы.

Экстракция ДНК проведена набором Qiagen DNA blood mini kit (Германия). Предварительно забранный биоматериал (кровь) была консервирован с помощью раствора Ficoll. Оценка длины теломер лейкоцитов проведена методом полимеразной цепной реакции в реальном времени (алгоритм Flow-fish). Статистический анализ выполнен с использованием программы IBM SPSS Statistics (версия 26,0 для Windows).

**РЕЗУЛЬТАТЫ:**

РЕЗУЛЬТАТЫ.

**1:**

1.Пациентки в физиологической менопаузе и на МГТ имели более высокий уровень ХС ЛПВП (p<0,006).

**2:**

2.Пациентки с ПНЯ наиболее склонны к повышению уровня креатинина сыворотки крови (p<0,001).

**3:**

3.Пациентки репродуктивного возраста без патологии имели достоверно наиболее высокую длину теломер (p<0,001).

**4:**

4.Уровень фолликулостимулирующего гормона умеренно отрицательно (ρ=-0,434) коррелирует с длиной теломер лейкоцитов у женщин (р<0,001).

**ЗАКЛЮЧЕНИЕ:**

ЗАКЛЮЧЕНИЕ. Группы пациенток с ПНЯ являются наиболее уязвимыми с точки зрения репликативных (длина теломер) и биохимических предикторов возраст-обусловленной патологии.

## Abstract (en)

**BACKGROUND:**

BACKGROUND: Estrogenic deficiency is the basic condition of human ageing that leads to hypergonadotropic hypogonadism. The existence of correlation between hypergonadotropic hypogonadism, replicative (leukocyte telomere length) and biochemical data is widely supposed among females with physiological (menopausal) and pathological (primary ovarian insufficiency) estrogenic deficiency is not unreasonable.

**AIM:**

AIM: To evaluate features of replicative (telomere length) and biochemical (metabolic syndrome) ageing markers among females with physiological (menopausal) and pathological (primary ovarian insufficiency) estrogenic deficiency.

**MATERIALS AND METHODS:**

MATERIALS AND METHODS: Research has been provided in collaboration between Endocrinology Research Centre of the Russian Ministry of Health and Lomonosov Moscow State University Medical Research and Educational Centre in the period since 10.01.2021 until 01.08.2022.

**110 females (20-75y:**

110 females (20-75y.o.) have participated in the present research.

**Group 1:**

Group 1: 26 females receiving menopausal hormonal therapy (MHT) ≥ 5 years with 0,5; 1; 2 mg estrogenic component.

**Group 2:**

Group 2: 27 females in physiological menopause without MHT

**Group 3:**

Group 3: 33 females with primary ovarian insufficiency and receiving sex-steroid replacement therapy.

**Group 4:**

Group 4: 24 healthy reproductive age females without sex-steroid replacement therapy.

Patients have undergone laboratory genetic (leucocyte telomere length), biochemical analyses.

DNA extraction — with Qiagen DNA blood mini kit (Germany). Biological material was cito conserved with Ficoll solution. Leukocyte telomere length — with real-time polymerase chain reaction PCR (Flow-fish).

Soft program IBM SPSS Statistics (version 26,0 for Windows) has been used for statical analysis.

**RESULTS:**

RESULTS.

**1:**

1.Menopausal females receiving MHT were inclined to highest HDL-P levels (p<0,006).

**2:**

2.Females with primary ovarian insufficiency were inclined to relatively highest serum creatinine level (p<0,001).

**3:**

3.Reproductive age females had relatively highest telomere length (p<0,001).

**4:**

4.FSH level correlates negatively and moderately (ρ= - 0,434) leukocyte telomere length (р<0,001) among females.

**CONCLUSION:**

CONCLUSION: Females with premature ovarian insufficiency are most sensible to ageing due to features of replicative and biochemical markers.

## ОБОСНОВАНИЕ

Проблема определения статистически достоверных маркеров женского старения и поиска anti-ageing терапии на протяжении многих столетий находится не только в зоне «infotainment» (с англ. «information» — информация + «entertainment» — развлечение), но и в центре внимания международного научно-медицинского сообщества. За последние 100 лет достижения научно-технического прогресса позволили добиться увеличения качества и длительности средней продолжительности жизни женщин, что отражено в соответствии с критериями шкалы Stages of Reproductive Aging Workshop (STRAW + 10) [1]. Одним из последствий такого рода смещения является то, что значительный процент женщин в развитых странах проводят практически треть своей жизни не только в состоянии менопаузы, но и в группе риска эстроген-дефицитной патологии (ишемическая болезнь сердца, артериальная гипертензия, деменция, остеопороз и т.д.) [2–4]. В свете современной парадигмы «здорового старения» ребром встает вопрос о разработке клинико-диагностического сопровождения пациенток с гипергонадотропным гипогонадизмом неятрогенного генеза (женщины в физиологической менопаузе, получающие/не получающие менопаузальную гормональную терапию (МГТ), женщины с преждевременной недостаточностью яичников (ПНЯ), здоровые женщины репродуктивного возраста) — маркера свершившегося «выключения» эндокринной функции яичников.

Из существующего на сегодняшний момент широкого спектра теорий старения наиболее многообещающей представляется синергизм эндокринной и теломеразной теорий старения in vivo. Согласно первой теории, интенсивность старения связана со снижением числа и аффинности эстрогеновых рецепторов ERα и ERβ на органах-мишенях [4]. Согласно другой теории, в основе старения лежит процесс дисфункции фермента теломеразы и укорочения теломер (от др.-греч. «τέλος» — конец + «μέρος» — часть) — концевых структур ДНК, имеющих в своем составе тандемные повторы нуклеотидных последовательностей TTAGGG на 3’-конце [5]

Кроме того, одним из аргументов в пользу существования оси «эстроген-теломераза-теломеры» является исследование, проведенное Gaydosh, Lauren и соавт. (2020) и доказавшее статистически значимое протективное влияние эстрогенов на длину теломер in vitro и in vivo [6].

Авторы настоящего исследования выдвигают гипотезу, в рамках которой утверждается взаимосвязь между выраженностью гипергонадотропного гипогонадизма и фактом приема МГТ/гормональную заместительную терапию (ГЗТ), с одной стороны, и длиной теломер — с другой.

## ЦЕЛЬ ИССЛЕДОВАНИЯ

Изучить особенности маркеров репликативного клеточного старения (длина теломер) и биохимических показателей у женщин с неятрогенными формами гипергонадотропного гипогонадизма.

## МАТЕРИАЛЫ И МЕТОДЫ

## Место и время проведения исследования

Исследование проведено на базе ФГБУ «НМИЦ эндокринологии» Минздрава России совместно с МНОЦ «МГУ им. М.В. Ломоносова» в период с 10.01.2021 г. по 01.08.2022 г.

## Изучаемые популяции

В одномоментном сравнительном исследовании всего приняли участие 110 женщин в возрасте 20–75 лет.

Группа 1: 26 пациенток в физиологической менопаузе, получавших МГТ ≥5 лет в дозе эстрогенового компонента 1; 2 мг.

Группа 2: 27 пациенток в физиологической менопаузе, не получавших МГТ.

Группа 3: 33 участницы с ПНЯ, получавших ГЗТ половыми стероидами.

Группа 4: 24 здоровые женщины репродуктивного возраста в возрасте 20–49 лет, не получающие ГЗТ.

## Изучаемые популяции пациентов

I.Пациентки в состоянии физиологической менопаузы, получающие МГТ более 5 лет (по данным медицинской документации), в т.ч. пациентки в дозе эстрогенового компонента 0,5; 1; 2 мг ≥15 лет (основная группа 1).

Критерии включения: пациентки женского пола паспортного возраста 50–75 лет, находящиеся в состоянии гипергонадотропного гипогонадизма вследствие физиологической менопаузы длительностью не менее 5 лет. Получение заместительной терапии половыми стероидами в дозе эстрогенового компонента 1; 2 мг более 5 лет — по данным медицинской документации. Все пациентки подписывали информированное согласие на проведение обследования и консультирование.

Критерии исключения

1.Наличие ятрогенной менопаузы в анамнезе.

1.1.После перенесенных хирургических вмешательств.

1.2.После перенесенной химиотерапии.

1.3.После перенесенной лучевой терапии.

1.4.После комбинированного лечения из вышеперечисленных выше методов.

2.Наличие сопутствующей патологии в анамнезе.

2.1.Эстроген-зависимые заболевания и патологические состояния (гиперпластические процессы эндометрия, миома матки, все формы эндометриоза).

2.2.Нарушения функции щитовидной железы.

2.3.Наличие официально задокументированных психических расстройств.

2.4.Наличие официально задокументированных злокачественных новообразований.

2.5.Нарушения углеводного обмена (нарушенная толерантность к глюкозе, нарушенная гликемия венозной плазмы натощак, сахарный диабет 1 и 2 типов).

2.6.Сердечно-сосудистые заболевания (ишемическая болезнь сердца (ИБС), острое нарушение мозгового кровообращения (ОНМК), тромбоэмболия легочной артерии (ТЭЛА)).

3.Другие физиологические состояния репродуктивной системы.

Способ формирования выборки — произвольный.

II.Участницы в состоянии физиологической менопаузы, без МГТ (группа сравнения 1).

Критерии включения: пациентки женского пола паспортного возраста 50–75 лет, находящиеся в состоянии гипергонадотропного гипогонадизма вследствие физиологической менопаузы длительностью не менее 5 лет; никогда не получавшие заместительную терапию половыми стероидами по данным медицинской документации. Все пациентки подписывали информированное согласие на проведение обследования и консультирование.

Критерии исключения

1.Наличие ятрогенной менопаузы в анамнезе.

1.1.После перенесенных хирургических вмешательств.

1.2.После перенесенной химиотерапии.

1.3.После перенесенной лучевой терапии.

1.4.После комбинированного лечения из вышеперечисленных выше методов.

2.Наличие сопутствующей патологии в анамнезе.

2.1.Наличие аутоиммунной менопаузы в анамнезе (в исходе первичной недостаточности яичников).

2.2.Эстроген-зависимые заболевания и патологические состояния (гиперпластические процессы эндометрия, миома матки, все формы эндометриоза).

2.3.Нарушения функции щитовидной железы.

2.4.Наличие официально задокументированных психических расстройств.

2.5.Наличие официально задокументированных злокачественных новообразований.

2.6.Нарушения углеводного обмена (нарушенная толерантность к глюкозе, нарушенная гликемия венозной плазмы натощак, сахарный диабет 1 и 2 типов).

2.7.Сердечно-сосудистые заболевания (ИБС, ОНМК, ТЭЛА).

3.Другие физиологические состояния репродуктивной системы.

Способ формирования выборки — произвольный.

III.Пациентки с установленным диагнозом «Преждевременная недостаточность яичников», получающие ГЗТ половыми стероидами >5 лет (основная группа 2).

Критерии включения: пациентки женского пола паспортного возраста <40 лет, находящиеся в состоянии аменореи длительностью не менее 5 лет; подтвержденный медицинской документацией факт получения заместительной терапии половыми стероидами в дозе эстрогенового компонента 1; 2 мг. Все пациентки подписывали информированное согласие на проведение обследования и консультирование.

Критерии исключения

1.Наличие ятрогенной менопаузы в анамнезе.

1.1.После перенесенных хирургических вмешательств.

1.2.После перенесенной химиотерапии.

1.3.После перенесенной лучевой терапии.

1.4.После комбинированного лечения из вышеперечисленных выше методов.

2.Наличие сопутствующей патологии в анамнезе.

2.1.Эстроген-зависимые заболевания и патологические состояния (гиперпластические процессы эндометрия, миома матки, все формы эндометриоза).

2.2.Нарушения функции щитовидной железы.

2.3.Наличие официально задокументированных психических расстройств.

2.4.Наличие официально задокументированных злокачественных новообразований.

2.5.Нарушения углеводного обмена (нарушенная толерантность к глюкозе, нарушенная гликемия венозной плазмы натощак, сахарный диабет 1 и 2 типов).

2.6.Сердечно-сосудистые заболевания (ИБС, ОНМК, ТЭЛА).

3.Другие физиологические состояния репродуктивной системы.

3.1.Беременность.

3.2.Период грудного вскармливания.

Способ формирования выборки — произвольный.

IV.Здоровые женщины репродуктивного возраста без заболеваний репродуктивной системы, не получающие ГЗТ (группа сравнения 2).

Критерии включения: пациентки женского пола паспортного возраста 20–49 лет с сохраненным менструальным циклом; уровень ФСГ в фолликулярную фазу — в пределах 2,0–11,6 МЕ/л, в лютеиновую фазу — в пределах 1,4–9,6 МЕ/л. Все пациентки подписывали информированное согласие на проведение обследования и консультирование.

Критерии исключения

1.Наличие физиологической менопаузы в анамнезе.

2.Наличие ятрогенной менопаузы в анамнезе.

2.1.После перенесенных хирургических вмешательств.

2.2.После перенесенной химиотерапии.

2.3.После перенесенной лучевой терапии.

2.4.После комбинированного лечения из вышеперечисленных выше методов.

3.Наличие сопутствующей патологии в анамнезе.

3.1.Наличие аутоиммунной менопаузы в анамнезе (в исходе первичной недостаточности яичников).

3.2.Эстроген-зависимые заболевания и патологические состояния (гиперплазия эндометрия, миома матки, все формы эндометриоза).

3.3.Нарушения функции щитовидной железы.

3.4.Наличие официально задокументированных психических расстройств.

3.5.Наличие официально задокументированных злокачественных новообразований.

3.6.Нарушения углеводного обмена (нарушенная толерантность к глюкозе, нарушенная гликемия венозной плазмы натощак, сахарный диабет 1 и 2 типов).

3.7.Сердечно-сосудистые заболевания (ИБС, ОНМК, ТЭЛА).

4.Другие физиологические состояния репродуктивной системы.

4.1.Беременность.

4.2.Период грудного вскармливания.

Способ формирования выборки — произвольный.

## Описание вмешательства

Пациенткам проведен лабораторный генетический (длина теломер лейкоцитов), биохимический анализы.

Экстракция ДНК проведена набором Qiagen DNA blood mini kit (Германия). Консервация биоматериала проводилась после забора cito с применением раствора Ficoll.

Оценка длины теломер лейкоцитов — методом полимеразной цепной реакции (ПЦР) в реальном времени (алгоритм Flow-fish).

## Дизайн исследования

Оригинальное активное одномоментное сравнительное.

## Статистический анализ

Статистическая обработка данных выполнена с помощью программы IBM SPSS Statistics (version 26,0 for Windows).

Количественные показатели оценивались на предмет соответствия нормальному распределению с помощью критерия Шапиро–Уилка.

Категориальные данные описывались с указанием абсолютных значений и процентных долей.

Сравнение процентных долей при анализе многопольных таблиц сопряженности выполнялось с помощью критерия хи-квадрат Пирсона.

Количественные показатели, имеющие нормальное распределение, описывались с помощью средних арифметических величин (M) и стандартных отклонений (SD), границ 95% доверительного интервала (95% ДИ). В случае отсутствия нормального распределения количественные данные описывались с помощью медианы (Me) и нижнего и верхнего квартилей [Q1–Q3].

Сравнение исследуемых групп по количественному показателю, имеющему нормальное распределение, выполнялось с помощью однофакторного дисперсионного анализа, апостериорные сравнения проводились с помощью критерия Тьюки.

Сравнение исследуемых групп по количественному показателю, распределение которого отличалось от нормального, выполнялось с помощью критерия Краскела–Уоллиса, апостериорные сравнения — с помощью критерия Данна с поправкой Холма.

Корреляционная связь оценивались с помощью коэффициента корреляции Спирмена.

## Этическая экспертиза

Протокол исследования был одобрен этическим комитетом ФГБУ «НМИЦ эндокринологии» Минздрава России (№11 от 22.07.2021).

## РЕЗУЛЬТАТЫ

1.Пациентки с ПНЯ наиболее склонны к повышению уровня креатинина сыворотки крови (69,7 [ 63,6–72,2], p<0,001).

2.Пациентки репродуктивного возраста без патологии в анамнезе имели достоверно наиболее высокую длину теломер (10,8 [ 10,0–13,1] кБ, p<0,001).

3.Уровень ФСГ умеренно отрицательно (ρ=-0,434) коррелирует с длиной теломер лейкоцитов у женщин (р<0,001).

Статистически значимое распределение пациентов из задействованных в исследовании групп по возрасту наглядно представлено в табл. 1 и на рис. 1. Наиболее молодыми пациентками являются девушки с ПНЯ.

**Table:** Таблица 1. Анализ возраста в исследуемых группахTable 1. Age analysis in the study groups * — различия показателей статистически значимы (p<0,05).

Показатель	Категории	Возраст, лет	p
Me	Q₁–Q₃	n
Исследуемые группы	группа 1	60	55–63	26	<0,001* pгр 3–гр 1<0,001 pгр 4–гр 1<0,001 pгр 5–гр 1<0,001 pгр 3–гр 2<0,001 pгр 4–гр 2<0,001 pгр 5–гр 2<0,001 pгр 5–гр 3=0,009 pгр 5–гр 4=0,014
группа 2	58	54–62	27
группа 3	36	29–39	33
группа 4	36	30–40	24

**Figure:**
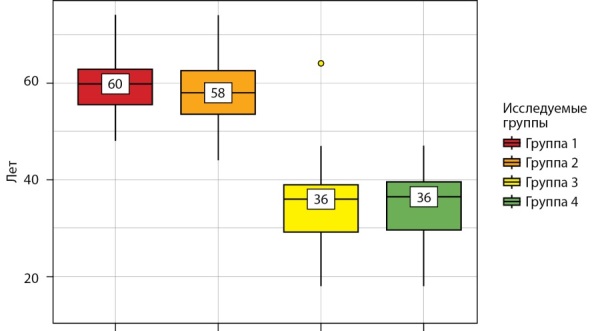
Рисунок 1. Анализ возраста в исследуемых группах.Figure 1. Age analysis in the study groups.

Статистически значимый возраст наступления физиологической менопаузы у пациенток соответствует общепопуляционным значениям. Представители группы 4 в связи с сохранной репродуктивной функцией в данном случае исключены из сравнения (табл. 2).

**Table:** Таблица 2. Распределение пациенток по возрасту наступления менопаузыTable 2. Distribution of patients by age of menopause * — различия показателей статистически значимы (p<0,05).

Показатель	Категории	Возраст менопаузы, лет	p
Me	Q₁–Q₃	n
Исследуемые группы	группа 1	49	46–52	26	<0,001*
группа 2	48	44–50	27
группа 3	30	22–33	33
группа 4	-	-	-

В соответствии с вышеуказанными данными наиболее высокая и статистически значимая длина теломер (10,8 [ 10,0–13,1] кБ, p<0,001) отмечается у пациенток репродуктивного возраста без патологии в анамнезе. На втором месте — пациентки с ПНЯ (10,0 [ 7,9–10,7] кБ, p<0,001). Значимо сопоставимая длина теломер (p<0,001) выявлена у пациенток в состоянии физиологической менопаузы, получающих/не получающих МГТ (табл. 3).

**Table:** Таблица 3. Распределение длины теломер по группам (кБ)Table 3. Distribution of telomere length by groups (kB) * — различия показателей статистически значимы (p<0,05).

Показатель	Категории	Длина теломер, кБ	p
Me	Q₁–Q₃	n
Исследуемые группы	группа 1	9,8	9,5–10,0	26	<0,001*
группа 2	9,8	9,6–10,3	27
группа 3	10,0	7,9–10,7	32
группа 4	10,8	10,0–13,1	24

**Figure:**
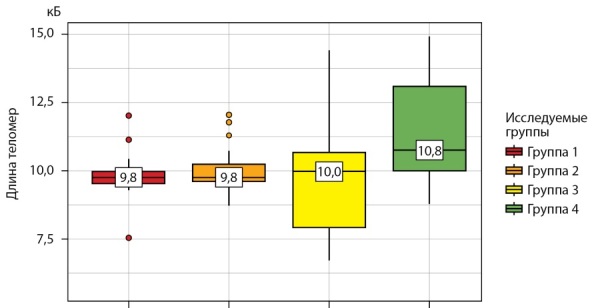
Рисунок 2. Распределение длины теломер по группам (кБ).Figure 2. Distribution of telomere length by groups (kB)

Согласно табл. 4, ни в одной группе пациенток с неятрогенным гипергонадотропным гипогонадизмом статистически значимых данных за распределение индекса массы тела так и не было получено (р=0,5).

**Table:** Таблица 4. Распределение показателй индекса массы тела по группамTable 4. Distribution of indicators of body mass index by groups

Показатель	Категории	ИМТ, кг/м2	p
Me	Q₁–Q₃	n
Исследуемые группы	группа 1	26	22–28	26	0,5
группа 2	26	22–28	27
группа 3	24	20–30	33
группа 4	22	20–27	24

Согласно табл. 5, у пациенток во всех группах отмечались статистически значимые сопоставимые показатели САД (р<0,001).

**Table:** Таблица 5. Распределение САД среди пациентокTable 5. Distribution of SBP among patients * — различия показателей статистически значимы (p<0,05).

Показатель	Категории	САД, мм рт.ст	p
M±SD	95% ДИ	n
Исследуемые группы	группа 1	127±12	122–132	26	<0,001*
группа 2	127±12	123–132	27
группа 3	130±14	125–135	33
группа 4	128±14	122–135	24

Пациентки в физиологической менопаузе продемонстрировали более высокую склонность к нарушениям углеводного обмена, что объясняется хронологическим возрастом (p<0,043) (табл. 6).

**Table:** Таблица 6. Наличие нарушенной гликемии венозной плазмы натощак в исследуемых группахTable 6. The presence of impaired fasting venous plasma glycemia in the study groups * — различия показателей статистически значимы (p<0,05).

Показатель	Категории	Нарушенная гликемия венозной плазмы натощак (≥6,1<7,0 ммоль/л)	p
Есть	Нет
Исследуемые группы	группа 1 (n=26)	3 (11,5)	23 (88,5)	0,043*
группа 2 (n=27)	3 (11,1)	24 (88,9)
группа 3 (n=33)	1 (3,0)	32 (97,0)
группа 4 (n=24)	0 (0,0)	24 (100,0)

Статистически значимой склонности к наличию гипотиреоза и соответствующему лабораторному смещению уровня ТТГ (p=0,9) не наблюдалось (p=0,8) ни в одной из групп пациенток (табл. 7, 27).

**Table:** Таблица 7. Распределение пациенток в зависимости от наличия гипотиреозаTable 7. Distribution of patients depending on the presence of hypothyroidism

Показатель	Категории	Гипотиреоз	p
Есть	Нет
Исследуемые группы	группа 1 (n=26)	5 (19,2)	21 (19,1)	0,8
группа 2 (n=27)	5 (19,2)	22 (20,0)
группа 3 (n=33)	8 (30,8)	25 (22,7)
группа 4 (n=24)	5 (19,2)	19 (17,3)

В рамках оценки показателей мочевины ни в одной из групп пациенток не обнаружено статистически значимых данных (p=0,08); отмечаются достоверные данные за повышение уровня креатинина при ПНЯ (p<0,001) (табл. 8, 9).

**Table:** Таблица 8. Сравнение уровня мочевины в группах пациентокTable 8. Comparison of urea levels in patient groups * — различия показателей статистически значимы (p<0,05).

Показатель	Категории	Мочевина	p
Me	Q₁–Q₃	n
Исследуемые группы	группа 1	6,0	4,9–7,9	26	0,08
группа 2	6,0	4,9–8,0	27
группа 3	5,6	5,0–7,2	33
группа 4	5,5	5,0–6,8	24

**Table:** Таблица 9. Сравнение уровня креатинина в исследуемых группахTable 9. Comparison of creatinine levels in the study groups * — различия показателей статистически значимы (p<0,05).

Показатель	Категории	Креатинин	p
Me	Q₁–Q₃	n
Исследуемые группы	группа 1	69,1	66,3–71,6	26	<0,001*
группа 2	69,2	66,4–71,8	27
группа 3	69,7	63,7–75,9	33
группа 4	67,3	63,6–72,2	24

По данным табл. 10, 11 статистически значимых различий по повышению уровня общего (p=0,2) и прямого (p=0,8) билирубина ни в одной из групп испытуемых обнаружено не было.

**Table:** Таблица 10. Сравнение уровня общего билирубина в исследуемых группахTable 10. Comparison of the level of total bilirubin in the study groups

Показатель	Категории	Билирубин общий	p
Me	Q₁–Q₃	n
Исследуемые группы	группа 1	10,7	9,0–13,1	26	0,2
группа 2	10,5	8,6–13,0	27
группа 3	10,0	8,2–14,6	33
группа 4	9,5	7,8–14,8	24

**Table:** Таблица 11. Сравнение уровня прямого билирубина в исследуемых группахTable 11. Comparison of the level of direct bilirubin in the study groups

Показатель	Категории	Билирубин прямой	p
Me	Q₁–Q₃	n
Исследуемые группы	группа 1	4,0	3,4–5,0	26	0,8
группа 2	3,9	3,5–5,0	27
группа 3	3,9	3,4–5,6	33
группа 4	3,9	2,6–5,2	24

По результатам, обозначенным в табл. 12, 13, 14, сравнения уровня трансаминаз (АЛТ, АСТ) и гамма-глутамилтранспептидазы (ГГТ) выявлена статистически значимая закономерность повышения АЛТ (p<0,001), у пациенток репродуктивного возраста без ГЗТ. Отмечается склонность к интерференции («взаимоналожению») разброса показателей АЛТ (p<0,001), ГГТ (p<0,001).

**Table:** Таблица 12. Сравнение уровня АЛТ в исследуемых группахTable 12. Comparison of ALT levels in the study groups * — различия показателей статистически значимы (p<0,05).

Показатель	Категории	АЛТ	p
Me	Q₁–Q₃	n
Исследуемые группы	группа 1	11,00	9,25–16,75	26	<0,001*
группа 2	11,00	9,50–16,50	27
группа 3	12,00	10,00–15,00	33
группа 4	14,00	10,00–18,00	24

**Table:** Таблица 13. Сравнение уровня АСТ в исследуемых группахTable 13. Comparison of AST levels in the study groups * — различия показателей статистически значимы (p<0,05).

Показатель	Категории	АСТ	p
Me	Q₁–Q₃	n
Исследуемые группы	группа 1	21,50	17,00–25,75	26	<0,001*
группа 2	22,00	17,00–25,50	27
группа 3	16,00	14,00–19,00	33
группа 4	17,00	15,75–19,00	24

**Table:** Таблица 14. Сравнение уровня ГГТ в исследуемых группахTable 14. Comparison of GGT levels in the study groups * — различия показателей статистически значимы (p<0,05).

Показатель	Категории	ГГТ	p
Me	Q₁–Q₃	n
Исследуемые группы	группа 1	13,50	12,00–18,00	26	<0,001*
группа 2	14,00	12,00–18,00	27
группа 3	16,00	14,00–20,00	33
группа 4	16,00	13,75–20,00	24

По данным оценки липидного профиля у пациенток репродуктивного возраста без ГЗТ отмечаются наиболее высокие и статистически значимые показатели триглицеридов (табл. 15, p<0,003), относительно более широкий интерквартильный разброс показателей уровня фосфатемии (табл. 21, p<0,001), наиболее низкие уровни витамина D (табл. 22, p<0,001) и калия (табл. 24, p=0,007). У пациенток в физиологической менопаузе и на МГТ отмечался более высокий уровень ХС ЛПВП (табл. 16, p=0,006*).

**Table:** Таблица 15. Сравнение уровня триглицеридов в исследуемых группахTable 15. Comparison of triglyceride levels in the study groups * — различия показателей статистически значимы (p<0,05).

Показатель	Категории	Триглицериды	p
Me	Q₁–Q₃	n
Исследуемые группы	группа 1	0,78	0,55–1,01	26	0,003*
группа 2	0,80	0,55–1,00	27
группа 3	0,78	0,59–0,98	33
группа 4	0,82	0,64–1,00	24

**Table:** Таблица 16. Сравнение уровня ХС ЛПВП в исследуемых группахTable 16. Comparison of HDL-C levels in the studied groups * — различия показателей статистически значимы (p<0,05).

Показатель	Категории	Холестерин ЛПВП	p
Me	Q₁–Q₃	n
Исследуемые группы	группа 1	1,9	1,4–2,2	26	0,006*
группа 2	1,9	1,4–2,1	27
группа 3	1,8	1,6–2,1	33
группа 4	1,8	1,6–2,5	24

По данным оценки антропометрических показателей (рост, вес) у пациенток во всех группах отмечалась статистически значимая интерференция («взаимоналожение») показателей интерквартильного размаха (табл. 29, 30, p<0,001).

Статистически значимых показателей уровня общего холестерина (табл. 17, p=0,3), ХС ЛПНП (табл. 18, p=0,1), общего/ионизированного кальция (табл. 19, 20; p=0,7/0,7), натрия (табл. 23, p=0,9), хлоридов (табл. 25, p=0,1), гликированного гемоглобина (табл. 28, p=0,4) в группах выявлено не было.

**Table:** Таблица 17. Сравнение уровня общего холестерина в исследуемых группахTable 17. Comparison of total cholesterol levels in the study groups

Показатель	Категории	Холестерин общий	p
Me	Q₁–Q₃	n
Исследуемые группы	группа 1	4,80	4,47–5,50	26	0,3
группа 2	4,81	4,50–5,47	27
группа 3	4,58	4,16–5,02	33
группа 4	4,86	4,55–5,25	24

**Table:** Таблица 18. Сравнение уровня ХС ЛПНП в исследуемых группахTable 18. Comparison of LDL-C levels in the study groups

Показатель	Категории	Холестерин ЛПНП	p
Me	Q₁–Q₃	n
Исследуемые группы	группа 1	2,5	2,3–3,4	26	0,1
группа 2	2,5	2,3–3,4	27
группа 3	2,6	2,1–3,0	33
группа 4	2,7	2,1–3,0	24

**Table:** Таблица 19. Сравнение уровня общего кальция в исследуемых группахTable 19. Comparison of total calcium levels in study groups

Показатель	Категории	Кальций общий	p
Me	Q₁–Q₃	n
Исследуемые группы	группа 1	2,28	2,20–2,41	26	0,7
группа 2	2,28	2,21–2,40	27
группа 3	2,31	2,27–2,36	33
группа 4	2,30	2,27–2,36	24

**Table:** Таблица 20. Сравнение уровня ионизированного кальция в исследуемых группахTable 20. Comparison of the level of ionized calcium in the study groups

Показатель	Категории	Кальций ионизированный	p
Me	Q₁–Q₃	n
Исследуемые группы	группа 1	1,08	1,05–1,13	26	0,7
группа 2	1,08	1,05–1,13	27
группа 3	1,08	1,05–1,11	33
группа 4	1,08	1,05–1,12	24

**Table:** Таблица 21. Сравнение уровня фосфора в исследуемых группахTable 21. Comparison of phosphorus levels in the study groups * — различия показателей статистически значимы (p<0,05).

Показатель	Категории	Фосфор	p
Me	Q₁–Q₃	n
Исследуемые группы	группа 1	1,10	0,96–1,23	26	<0,001*
группа 2	1,12	0,96–1,23	27
группа 3	1,17	0,97–1,29	33
группа 4	1,17	1,10–1,31	24

**Table:** Таблица 22. Сравнение уровня витамина D в исследуемых группахTable 22. Comparison of vitamin D levels in study groups * — различия показателей статистически значимы (p<0,05).

Показатель	Категории	Витамин D	p
Me	Q₁–Q₃	n
Исследуемые группы	группа 1	41,85	30,00–44,30	26	<0,001*
группа 2	42,00	30,10–44,40	27
группа 3	30,80	23,30–41,90	33
группа 4	27,55	22,18–35,50	24

**Table:** Таблица 23. Сравнение уровня натрия в исследуемых группахTable 23. Comparison of sodium levels in study groups

Показатель	Категории	Натрий	p
M ± SD	95% ДИ	n
Исследуемые группы	группа 1	138,5 ± 4,0	136,9–140,2	26	0,9
группа 2	138,5 ± 3,9	137,0–140,1	27
группа 3	138,1 ± 3,0	137,1–139,2	33
группа 4	138,1 ± 2,7	137,0–139,3	24

**Table:** Таблица 24. Сравнение уровня калия в исследуемых группахTable 24. Comparison of potassium levels in the study groups * — различия показателей статистически значимы (p<0,05).

Показатель	Категории	Калий	p
Me	Q₁–Q₃	n
Исследуемые группы	группа 1	4,7	4,0–5,8	26	0,007*
группа 2	4,8	4,0–6,3	27
группа 3	4,6	4,0–5,0	33
группа 4	4,4	4,1–5,1	24

**Table:** Таблица 25. Сравнение уровня хлоридов в исследуемых группахTable 25. Comparison of chloride levels in study groups

Показатель	Категории	Хлориды	p
Me	Q₁–Q₃	n
Исследуемые группы	группа 1	104,0	102,2–106,0	26	0,1
группа 2	104,0	102,5–106,0	27
группа 3	105,0	102,0–106,0	32
группа 4	105,0	103,0–106,0	23

С целью изучения выраженности гипергонадотропного гипогонадизма в группе оценивался уровень ФСГ (табл. 26), повышение которого является самым первым маркером наступления менопаузы. По результатам оценки достоверно выявлено, что наиболее высокий уровень ФСГ наблюдался у пациенток с ПНЯ (p<0,001). Обращает на себя внимание статистически значимое снижение уровня ФСГ у пациенток в физиологической менопаузе (p<0,001), получающих заместительную терапию половыми стероидами, в отличие от группы без подобного рода терапии (p<0,001).

**Table:** Таблица 26. Сравнение уровня ФСГ в исследуемых группахTable 26. Comparison of FSH levels in study groups * — различия показателей статистически значимы (p<0,05).

Показатель	Категории	ФСГ	p
Me	Q₁–Q₃	n
Исследуемые группы	группа 1	47,00	41,25–80,75	26	<0,001*
группа 2	72,00	46,50–82,00	27
группа 3	92,00	91,00–95,00	33
группа 4	5,85	4,75–8,55	24

**Table:** Таблица 27. Сравнение уровня ТТГ в исследуемых группахTable 27. Comparison of TSH levels in the study groups

Показатель	Категории	ТТГ	p
Me	Q₁–Q₃	n
Исследуемые группы	группа 1	2,3	1,1–2,7	26	0,9
группа 2	2,3	1,0–2,7	27
группа 3	2,1	1,4–3,6	33
группа 4	1,9	1,4–2,9	24

**Table:** Таблица 28. Анализ гликированного гемоглобина в исследуемых группахTable 28. Analysis of glycated hemoglobin in the study groups

Показатель	Категории	Гликированный гемоглобин (HbA1c)	p
M±SD	95% ДИ	n
Исследуемые группы	группа 1	5,5±0,4	5,4–5,7	26	0,4
группа 2	5,6±0,4	5,4–5,7	27
группа 3	5,4±0,4	5,3–5,5	33
группа 4	5,4±0,4	5,2–5,5	24

**Table:** Таблица 29. Анализ роста в исследуемых группахTable 29. Analysis of growth in the study groups * — различия показателей статистически значимы (p<0,05).

Показатель	Категории	Рост, см	p
M±SD	95% ДИ	n
Исследуемые группы	группа 1	167±9	163–170	26	<0,001*
группа 2	167±9	163–171	27
группа 3	168±9	165–172	33
группа 4	168±10	164–173	24

**Table:** Таблица 30. Вес, кг в исследуемых группахTable 30. Weight, kg in the study groups * — различия показателей статистически значимы (p<0,05).

Показатель	Категории	Вес, кг	p
M±SD	95% ДИ	n
Исследуемые группы	группа 1	71,1±14,4	65,3–77,0	26	<0,001*
группа 2	70,6±14,4	64,9–76,3	27
группа 3	69,9±13,8	65,0–74,8	33
группа 4	67,1±12,7	61,8–72,4	24

Между уровнем ФСГ и длиной теломер лейкоцитов была установлена статистически достоверная умеренная обратная корреляционная связь (ρ=-0,434, p<0,001), что демонстрирует снижение репликативного потенциала клетки в исходе развития отрицательного влияния гипергонадотропного гипогонадизма (табл. 31). Чем выше значения ФСГ, тем короче длина теломер.

**Table:** Таблица 31. Корреляционный анализ ФГС и длины теломерTable 31. Correlation analysis of FGS and telomere length * — различия показателей статистически значимы (p<0,05).

Показатель	Характеристика корреляционной связи
ρ	Теснота связи по шкале Чеддока	p
ФСГ — Длина теломер	-0,434	Умеренная	<0,001*

## ОБСУЖДЕНИЕ

## Репрезентативность выборок

В данном исследовании задействован относительно малый и неоднородный объем выборки как в контрольной, так и в основной группах пациенток. Сниженный уровень репрезентативности объясняется относительно редкой в популяции частотой наличия ПНЯ, с одной стороны, и высокой долей пациенток в репродуктивном возрасте, постменопаузе — с другой. На объем и неоднородность выборки неблагоприятным образом повлияли финансово-техническая ограниченность исследования, принцип строгого соблюдения критериев отбора, включения и исключения (по данным анамнеза и катамнеза) во избежание искажения конечных результатов.

## Сопоставление с другими публикациями

Патология теломер в виде их укорочения в сочетании с несбалансированной лайонизацией хромосомы Х (т.е. инактивацией Х-хромосом в эмбриональном периоде), по данным Miranda-Furtado C.L. и соавт. (2018) [7], статистически значимо обнаруживается (экспериментальная группа 0,93±0,23 кБ; контроль 1,07±0,2) у пациенток с клинически и лабораторно подтвержденной ПНЯ.

В то же время, по данным исследования «случай-контроль», проведенного в 2017 г. на базе отделения ВРТ университетской клиники г. Анкары (Турция), между пациентками с/без ПНЯ в анамнезе не было обнаружено статистически значимой разницы в уровне активности фермента теломеразы в сыворотке [8]; подобного рода противоречивые и неоднозначные исследования свидетельствуют в пользу необходимости изучения репликативной функции в будущем исключительно по данным биопсии яичников на фоне/без приема ЗГТ.

## Клиническая значимость результатов

Неятрогенный гипергонадотропный гипогонадизм у женщин — собирательное наднозологичное понятие, объединяющее пациенток с естественным (т.е. не индуцированным извне) развитием менопаузы вследствие физиологичных (естественная менопауза) и аутоиммунных причин (ПНЯ). Чем раньше в онтогенезе у женщины формируются предпосылки для менопаузы (ПНЯ — в возрасте до 40 лет), тем больший риск развития коморбидности грозит пациентке последующим снижением качества и продолжительности жизни. Помимо наглядного доказательства обратной корреляции между уровнем ФСГ и длиной теломер, данное исследование демонстрирует важность индивидуального подбора, разработки и назначения оптимальных схем при МГТ и ЗГТ половыми стероидами.

## Ограничения исследования

Основными факторами, ограничивающими масштаб и внешнюю валидность данного исследования, являлись относительная узость и неоднородность выборки вследствие крупной финансово затратной составляющей и относительной ограниченности допустимых временных рамок.

## Направления дальнейших исследований

В качестве логически оправданного продолжения вышеупомянутой идеи нами предполагается проведение в перспективе слепых плацебо-контролируемых рандомизированных клинических исследований, неотъемлемой частью которых должны стать проведение биопсии (пункции) яичников, определение длины теломер как до, так и после многолетнего приема МГТ/ГЗТ более широкими выборками пациенток из вышеописанных 5 категорий (на более обширных выборках). Осуществление такого рода дизайна на практике пока что остается затруднительным с этической и финансово-технической точек зрения.

## ЗАКЛЮЧЕНИЕ

Результаты данного исследования позволяют положительно оценить значение определения уровня ФСГ и длины теломер лейкоцитов как предиктора коморбидности и продолжительности жизни. Наличие, по данным исследований, эстрогенного дефицита in vivo неблагоприятным образом влияет на жизненный прогноз пациенток за есчет ускоренного развития многогранной (по разным органам и системам) эстроген-дефицитной коморбидности, потери трудоспособности и шансов на здоровое долголетие. Вопрос о разработке мер по персонализированной диагностике и терапии пациенток неятрогенного гипергонадотропного гипогонадизма обещает стать одним из наиболее насущных в ближайшие десятилетия.

## ДОПОЛНИТЕЛЬНАЯ ИНФОРМАЦИЯ

Источник финансирования. Исследование проводится в рамках Государственного задания: «Влияние эпигенетических факторов на течение менопаузы у женщин с эндокринопатиями аутоиммунного генеза в рамках формирования модели “здорового старения”», регистрационный номер АААА-121030100033-4.

Конфликт интересов. Авторы декларируют отсутствие явных и потенциальных конфликтов интересов, связанных с публикацией настоящей статьи.

Участие авторов. Михеев Р.К. — вклад по критерию 1, по критерию 2; Андреева Е.Н. — вклад по критерию 1, по критерию 2; Григорян О.Р. — вклад по критерию 1, по критерию 2; Шереметьева Е.В. — вклад по критерию 1, по критерию 2; Абсатарова Ю.С.— вклад по критерию 1, по критерию 2; Волеводз Н.Н. — вклад по критерию 1, по критерию 2; Логинова Е.В. — вклад по критерию 1, по критерию 2.

Все авторы одобрили финальную версию статьи перед публикацией, выразили согласие нести ответственность за все аспекты работы, подразумевающую надлежащее изучение и решение вопросов, связанных с точностью или добросовестностью любой части работы.
